# Lemierre’s syndrome presenting with arterial and Central nervous system involvement

**DOI:** 10.1093/bjrcr/uaae026

**Published:** 2024-08-08

**Authors:** Brandon Simons, Mason Williams, Laura Hayes, Kanika Gupta, Tushar Chandra

**Affiliations:** University of Central Florida College of Medicine, Orlando, FL 32827, United States; University of Central Florida College of Medicine, Orlando, FL 32827, United States; University of Central Florida College of Medicine, Orlando, FL 32827, United States; Nemours Children’s Hospital Department of Radiology, Orlando, FL 32827, United States; University of Arizona Medical Center—University Medical Center Tucson Department of Medical Imaging, Tucson, AZ 85724, United States; University of Central Florida College of Medicine, Orlando, FL 32827, United States; Nemours Children’s Hospital Department of Radiology, Orlando, FL 32827, United States

**Keywords:** Lemierre’s syndrome, meningitis, abscess, venous thrombosis, stroke, carotid

## Abstract

A 17-year-old male presented with acute onset right-sided facial swelling, trismus, pharyngitis, and sepsis. An initial CT abdomen and pelvis revealed multifocal bilateral nodular cavitary lung lesions. CT soft tissue neck with contrast demonstrated a parapharyngeal abscess and thrombophlebitis of the right internal jugular vein. The patient was subsequently diagnosed with Lemierre’s syndrome. On the following day, the patient’s neurological status markedly declined. Brain MRI/MRA/MRV showed right internal carotid artery narrowing, multiple areas of acute and subacute infarctions secondary to vasculitis, meningitis, venous sinus thrombosis, and intracerebral abscesses. Workup for primary causes of intracranial vasculitis was negative. Although commonly presented as venous disease, this case highlights a rare presentation of Lemierre’s syndrome with arterial involvement and significant intracranial complications. Clinicians should consider vasculitis and central nervous system involvement as potential complications of Lemierre’s syndrome rather than searching for separate aetiologies.

## Clinical presentation

A 17-year-old male with a medical history of controlled asthma presented to the emergency department with a chief complaint of worsening right-sided facial swelling over the past 2 days. Two weeks prior, the patient noticed a sore throat, myalgia, and fever. He tested positive for the flu and was prescribed Tamiflu. Over the next week, his symptoms worsened along with right cheek swelling, right ear pain making it difficult to speak, and difficulty fully opening his mouth. The patient presented to his primary care physician where Tamiflu was discontinued, and he was started on amoxicillin and steroids. The swelling of his face worsened with his eye swollen shut. He developed headache, cough, dizziness, vomiting, and diarrhoea.

On presentation to the emergency department, the patient was febrile (103.1 °F), saturating 95% O_2_ on room air, hypertensive (150/82 mm Hg), tachypneic (37 breaths/min), and had a pulse of 87 beats/min. On physical examination he was in moderate distress and diaphoretic. Eye examination revealed right-sided proptosis, upper and lower eyelid oedema, and exophthalmos with icteric conjunctiva. There was significant right-sided facial swelling with tenderness, and he was unable to open his mouth for proper inspection. Diminished breath sounds were appreciated at the right lower lobe of the lung on auscultation. He had minimal peripheral oedema and no rashes or lesions were noted on the extremities.

His labs upon admission revealed leucocytosis of 25.61 K/µL (4.0-10.5 K/µL) with neutrophils of 88.5% (50.0%-70.0%), thrombocytopenia of 90 K/µL (140-450 K/µL), coagulopathy with a PT of 27.3 s (11-13.5 s), PTT of 33 s (25-35 s), international normalized ratio of 2.4 (<1.1), d-dimer of 707.0 ng/mL (0.0-243.0 ng/mL) and a fibrinogen of 601 mg/dL (200-400 mg/dL), transaminitis with an aspartate aminotransferase of 304 U/L (0-32 U/L) and alanine aminotransferase of 286 U/L (0-33 U/L), alkaline phosphatase of 169 U/L (30-115 U/L), hypoalbuminemia of 2.10 g/dL (3.00-5.00 g/dL), slight hyponatremia of 132 mEq/L (135-145 mEq/L), procalcitonin of 54.62 ng/mL (0.0-18.0 ng/mL), CRP of 29.1 mg/dL, lactic acid of 4.60 mmol/L (0.50-1.90 mmol/L), and lactate dehydrogenase of 445 U/L (100-240 U/L).

Initial workup for group A Streptococcus, influenzas A and B, and SARS-CoV were negative. Elevated Epstein-Barr virus IgG levels were detected indicating past infection. A respiratory pathogen panel detected rhinovirus/enterovirus. An initial chest X-ray demonstrated no acute cardiopulmonary process. CT abdomen and pelvis revealed multifocal bilateral consolidative nodular cavitations of the lungs without pleural effusion. The remainder of CT the scan was unremarkable. The patient was transferred to inpatient care and started on broad spectrum antibiotic coverage.

## Imaging findings

**Figure 1.**
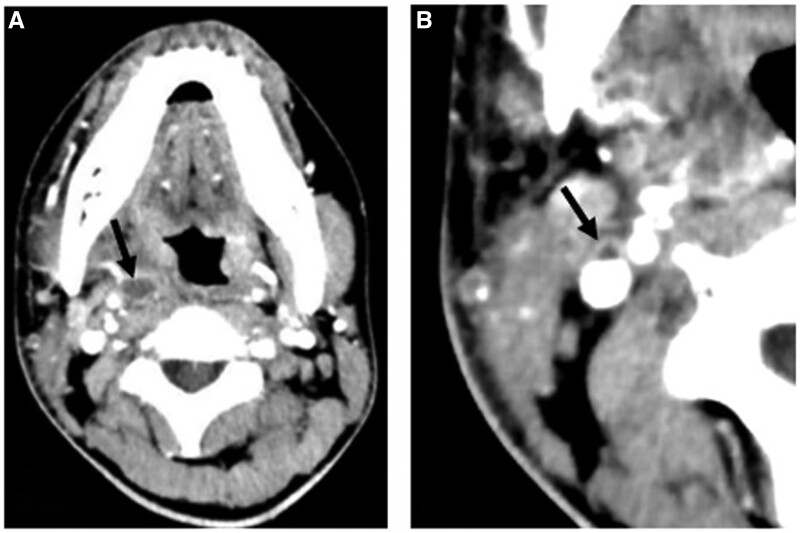
CT soft tissue neck with contrast (on the day of admission). Axial images from CT soft tissue neck with contrast demonstrating an abscess (arrow in A) in the right parapharyngeal space and an enhancing non-occlusive thrombus (arrow in B) in a small vein draining into the right internal jugular vein with vascular wall enhancement.

**Figure 2.**
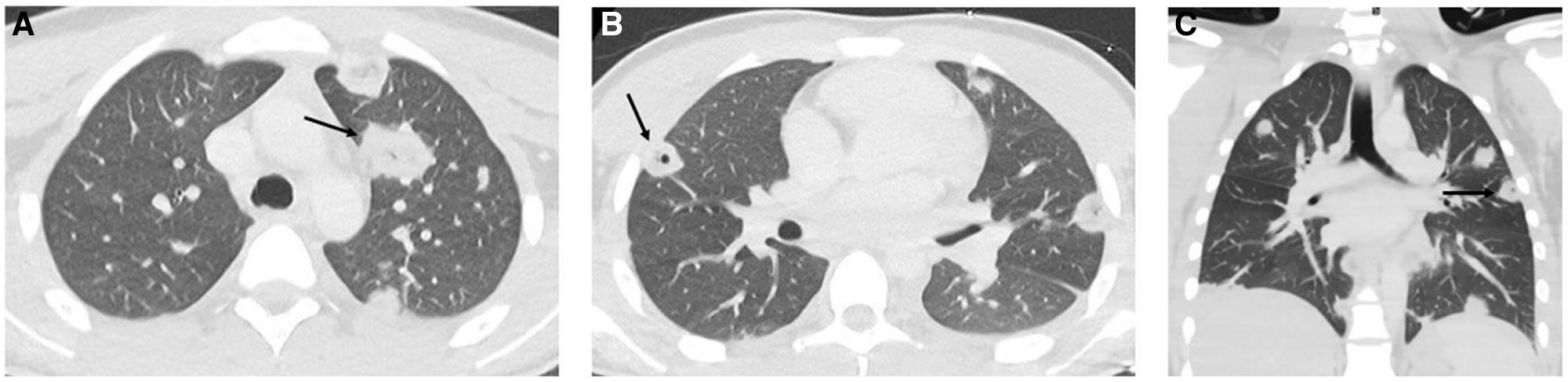
CT Chest with contrast (on the day of admission). Images from CT chest demonstrating large pulmonary nodules with spiculated margins and some with central cavitation (arrows).

The diagnosis of Lemierre’s syndrome was confirmed after cultures were noted to be growing *Fusobacterium necrophorum*, CT neck revealed a right parapharyngeal abscess with thrombophlebitis of the right internal jugular vein (Figure 1), and CT chest demonstrated bilateral septic pulmonary nodules (Figure 2).

**Figure 3.**
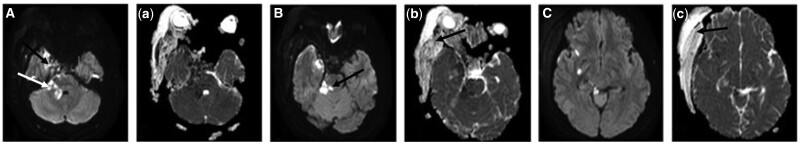
MRI Brain with and without contrast (1 day after admission). Diffusion-weighted imaging (uppercase) and apparent diffusion coefficient (lowercase) demonstrating multiple acute/subacute infarcts in the right medial temporal lobe (black arrow in image A), anteromedial aspect of the right cerebellum (white arrow in image A), and dorsolateral aspect of the right brainstem (arrow in image B). Also note soft tissue swelling on the right side of the face (arrows in images b and c).

Over the next 24 h the patient experienced worsening neurological status with increased confusion, aphasia, left facial droop, and left-sided hemiplegia. The patient was still able to follow simple commands. CT-STAT was unremarkable. MRI Brain revealed multiple focal areas of acute/subacute infarction involving the anterior and medial aspect of right temporal lobe, dorsal aspect of the midbrain and pons on the right side, right middle cerebral peduncle, medial aspect of right cerebellum, and the right external capsule and insula (Figure 3).

**Figure 4.**
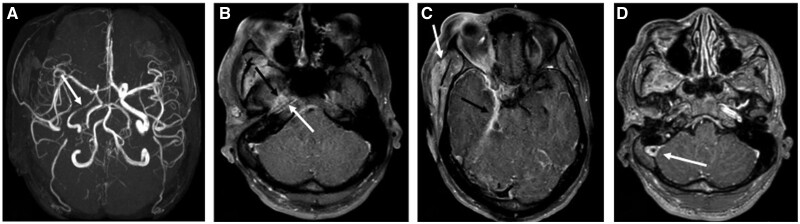
MR angiogram, MRI brain, and MR venogram (1 day after admission). (A) 3D time-of-flight MR angiogram demonstrating diffuse narrowing of the right internal carotid artery (white arrow). (B) Post-contrast T1 fat saturated image demonstrating prominent, abnormal enhancement along the wall of the petrous segment of right internal carotid artery (white arrow) and prominent meningeal and parenchymal enhancement is also seen along the adjacent temporal lobe (black arrow). (C) Post-contrast T1 fat saturated image revealing abnormal thick dural enhancement at the medial aspect of the right middle cranial fossa and the right tentorium (black arrow) and swelling and enhancement of the right temporalis muscle along with the overlying soft tissue (white arrow). (D) MR Venogram demonstrating central filling defect within the right sigmoid sinus (white arrow) in keeping with non-occlusive thrombus.

**Figure 5.**
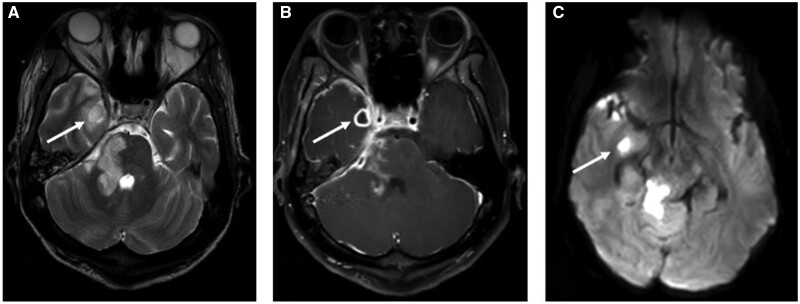
Brain MRI (8 days after admission). (A) T2 weighted image demonstrating soft tissue swelling on the right face, right sided proptosis, and a parenchymal lesion at the medical aspect of the right temporal lobe in keeping with an early abscess. (B and C) Post contrast T1 image and DWI demonstrating right medial temporal lobe abscess.

The underlying aetiology of the infarcts in the right middle cerebral artery territory was likely active vasculitis as noted by the abnormal enhancement of the vascular wall of the internal carotid artery and to a lesser extent middle cerebral arteries on the MR angiogram (Figure 4). To evaluate for primary vasculitis, ANCA, Russell viper venom time, dsDNA, ANA, and lupus anticoagulant were ordered with negative results. There was also evidence of meningitis, post septal cellulitis, and swelling of the right temporalis (Figure 4). Repeat neuroimaging 8 days after admission revealed the formation of an abscess in the right medial temporal lobe (Figure 5).

## Treatment, outcome, follow-up

On the day of admission, the patient was treated with vancomycin, metronidazole, and ceftriaxone. Following the diagnosis of Lemierre’s syndrome, the patient was switched to metronidazole and ceftriaxone therapy with heparin IV infusion. The patient received 2 doses of IVIG early in the hospital course and methylprednisolone for 4 days due to concern for an autoimmune process. Two weeks post-stroke the patient remained hemiplegic at the left upper and lower extremities and improved comprehension and alertness. Brain MRI 2 weeks after admission demonstrated no significant change in the size of the previous intracerebral abscess and the development of a separate abscess in the medial aspect of the right temporal lobe. Antibiotics and anticoagulation were continued for 6 weeks. During this time, the patient demonstrated improvement in serial neurological examinations and participated in multidisciplinary rehabilitation. At 6 weeks, the patient was able to follow commands and had slight improvement in left upper extremity weakness but still displayed dense left lower extremity hemiplegia. The patient was discharged after 13 weeks with significant neurological deficits including: moderate dysarthria, left upper extremity weakness, and inability to independently ambulate secondary to left lower extremity hemiplegia.

## Discussion

Lemierre’s syndrome is typically characterized by oropharyngeal infection progressing to septic thrombophlebitis of the internal jugular vein over the course of a couple weeks. *Fusobacterium necrophorum*, a native member of the normal oropharyngeal flora, is the most common causative pathogen.^1^ Complications that arise from septic embolization occur primarily in the lungs, in up to 97% of cases, followed by the large joints.^2–4^ Less common manifestations include osteomyelitis, endocarditis, pericarditis, and abscesses to the liver, spleen, and kidneys.^5^

Lemierre’s syndrome is most often confined to the internal jugular vein, and extension into the adjacent arteries is found in only 7.7% of cases. Among the cases with arterial presentations 38.2% involved stroke and 34.5% involved carotid stenosis. Patients with arterial or cardiac complications had a higher mortality (12%) compared to purely venous involvement (3.3%). The leading hypothesis of arterial involvement pathogenesis in Lemierre’s syndrome is direct invasion of the internal carotid from the internal jugular vein as both vessels are located within the carotid sheath. Some suggest the thicker arterial wall offers protection against infection accounting for the less common arterial involvement.^6^

Central nervous system complications are uncommon but may include brain abscesses, epidural abscesses, meningitis, subdural empyema, cerebral venous thrombosis, and strokes.^7^

Empiric therapy includes coverage of *F. necrophorum* and oral *streptococci* with piperacillin-tazobactam, a carbapenem, or ceftriaxone plus metronidazole. Once *F. necrophorum* is isolated, metronidazole monotherapy is administered for a minimum of 4 weeks. *F. necrophorum* has antibiotic resistance of about 2%.^8^ To date, there are no clear guidelines as to whether anticoagulation reduces thrombus growth or embolic events in Lemierre’s syndrome due to limited available data. However, the increased mortality with arterial involvement suggests anticoagulation may be warranted.^6^

This is a rare case of Lemierre’s syndrome reported with the combination of right internal carotid narrowing, multifocal areas of right hemisphere infarction secondary to vasculitis, cavernous venous thrombosis, meningitis, and an intracranial abscess. This case highlights the importance of considering vasculitis with intracranial complications as secondary to rare arterial involvement in Lemierre’s disease rather than seeking alternative causes of primary vasculitis and central nervous system involvement.

## Learning points

- Although commonly presenting as a venous thrombophlebitis, Lemierre’s syndrome may manifest with arterial involvement and various intracranial complications.
- Consider intracranial arterial vasculitis as secondary to Lemierre’s syndrome rather than evaluating for alternative causes of primary vasculitis.
- Consider central nervous system involvement as complications of Lemierre’s syndrome.
